# Association Between Antipsychotic Medication Use and Diabetes

**DOI:** 10.1007/s11892-019-1220-8

**Published:** 2019-09-02

**Authors:** Richard I. G. Holt

**Affiliations:** 1 Human Development and Health, Faculty of Medicine, University of Southampton, Southampton, UK; 2 University Hospital Southampton NHS Trust, The IDS Building (MP887), Southampton General Hospital, Tremona Road, Southampton, SO16 6YD UK

**Keywords:** Antipsychotics, Diabetes, Weight gain, Insulin resistance, β-cell dysfunction

## Abstract

**Purpose of Review:**

The prevalence of diabetes is 2–3-fold higher in people with severe mental illness than the general population. There are concerns that antipsychotics increase the risk of diabetes. This review will examine the latest epidemiological studies linking antipsychotics and diabetes, as well as the mechanisms underlying the association and the clinical implications to minimise the impact of antipsychotics on metabolic health.

**Recent Findings:**

Although there is an increased risk of diabetes in people with first-episode psychosis, the prevalence increases rapidly after antipsychotics are started. Antipsychotics likely increase the risk of diabetes through weight gain and directly by adversely affecting insulin sensitivity and secretion.

**Summary:**

It is important to implement measures to prevent diabetes, to screen for diabetes to ensure prompt diagnosis and to provide effective diabetes care. Further research is needed to understand how antipsychotics cause diabetes and to improve the clinical management of diabetes in people with severe mental illness.

## Introduction

The prevalence of diabetes is ~ 10% among people taking antipsychotics, which is 2–3-fold higher than the general population [1, 2]. Diabetes occurs at an earlier age and acute metabolic emergencies and diabetes complications have a greater impact in people with severe mental illness. The reasons underlying the increased rates of diabetes are multifactorial but there are concerns that antipsychotics play a role in the aetiology. This review will describe how these concerns emerged, the difficulties in determining causality followed by the evidence linking antipsychotics to the development of diabetes. Finally, I will discuss the clinical implications to minimise the impact of antipsychotics on metabolic health.

## History of Antipsychotics

The discovery of antipsychotics 70 years ago transformed the lives of people with psychosis. Prior to their availability, people with psychotic illnesses could expect to spend most of their lives in psychiatric asylums with treatment limited to sedation, electroconvulsive therapy, insulin-induced hypoglycaemic coma or frontal lobotomy. Like many discoveries in medicine, the therapeutic potential of antipsychotics was found serendipitously [3]. Promethazine, a phenothiazine antipsychotic, was first used in 1949 in people undergoing surgery to prevent shock by a French army surgeon who recognised that agitated and anxious patients became calm after its use. Chlorpromazine, a chlorinated phenothiazine, was used with success for the first time in 1952 by a person with mania. Over the next decade, psychiatrists began to incorporate chlorpromazine into clinical practice and with time, other phenothiazines, including trifluperazine, thioridazine and fluphenazine, were introduced.

A second class of antipsychotics, the butyrophenones, was developed in the late 1950s [4]. Haloperidol was initially synthesised as an analgesic but animal experiments demonstrated its antipsychotic effects. Collectively, the phenothiazine and butyrophenones are known as first-generation or ‘typical’ antipsychotics. Although their precise mechanisms of action remain uncertain, the major antipsychotic effect occurs by antagonising dopamine D_2_ receptors in the mesolimbic and mesocortical system of the brain (Table 1) [5]. First-generation antipsychotics also antagonise dopamine receptors in the nigrostriatal and tuberinfundibular system causing extrapyramidal movement disorders and hyperprolactinaemia respectively.

**Table 1:** Risk of weight gain, diabetes and receptor affinities of selected first- and second-generation antipsychotics

	Risk of weight gain	Risk of diabetes*	D_2_ dopamine	5HT_2c_ serotonin	5HT_1a_ serotonin	M_3_ muscarinic	α_2_ adrenergic	H_1_ histamine
Role in weight regulation			✓	✓				✓
Role in insulin secretion			✓		✓	✓	✓	
First-generation antipsychotic								
Chlorpromazine	+++	+++	++++	++++	+	++++	+	++++
Perphenazine	+	+	++++	++++	+	+	+	+++
Haloperidol	++	+	++++	++	−	+	+	+/−
Second-generation antipsychotic								
Clozapine	+++	+++	+++	+++	++	+++	++	+++
Olanzapine	+++	+++	+	+++	+	+++	+	+++
Quetiapine	++	++	+	+	+	+	+++	++
Risperidone	++	++	+++	++++	++	−	++++	++
Ziprasidone	+	+	+++	++++	++++	−	++	+
Aripiprazole	+	+	++++	+++	++++	−	++	+
Paliperidone	++	+	+++	++++	+	−	+++	++
Lurasidone	+	+	++++	++	++++	−	N/A	−

A higher risk of diabetes and weight gain is associated with high M_3_ Muscarinic and H_1_ Histamine receptor affinity

*Relative to other antipsychotics. Not all the risk of diabetes or weight gain is related to the antipsychotic

Once the side effects of the first-generation antipsychotics became apparent, work began to develop antipsychotics with a lower propensity to cause movement disorders. The first second-generation antipsychotic was clozapine, which was synthesised in 1958, and is based on the chemical structure of the tricyclic antidepressant imipramine [6]. Although highly effective, the manufacturers voluntarily withdrew clozapine in 1975 after reports of several deaths from agranulocytosis. The US Food and Drug Administration re-approved the drug in 2002 after trials showed conclusively that clozapine was more effective than other antipsychotics in the management of treatment-resistant schizophrenia. It now appears on the World Health Organization List of Essential Medicines. Following clozapine came a number of other second-generation antipsychotics in the 1990s and early 2000s including risperidone (approved 1993), olanzapine (1996), quetiapine (1997), ziprasidone (2001) and aripiprazole (2002).

The second-generation antipsychotics have a lower affinity for the D_2_ receptors, with the exception of aripiprazole, which is a partial D_2_ agonist (Table 1). As well as the action on D_2_ receptors, 5HT_2c_ antagonism and, in some cases, 5HT_1a_ agonism are thought to mediate the antipsychotic effect [7]. They cause less extrapyramidal symptoms because 5HT_2a_ receptor antagonism increases dopaminergic neurotransmission in the nigrostriatal pathway. In common with the first-generation antipsychotics, they have variable effects on muscarinic, histamine and adrenergic receptors.

Antipsychotics are approved for the treatment of schizophrenia and bipolar disorder but off-label use is widespread for conditions including obsessive–compulsive disorder, personality disorders, post-traumatic stress disorder, Tourette’s syndrome and anxiety.

As the use of the second-generation antipsychotics rose, reports of substantial weight gain, diabetes and dyslipidaemia began to emerge. In response, in 2003, the American Diabetes Association, American Psychiatric Association, American Association of Clinical Endocrinologists and American Association for the Study of Obesity convened a conference to discuss the risk of weight gain and diabetes in people taking antipsychotics [8]. The experts concluded that antipsychotics increased risk for obesity, diabetes and dyslipidaemia, with the risk of metabolic abnormalities being closely aligned to the degree of weight gain. Today, more than 15 years and several lawsuits later, the consensus remains that antipsychotics increase the risk of diabetes but the precise mechanisms involved require further elucidation.

## Challenges in Determining Causality

**Fig. 1:**
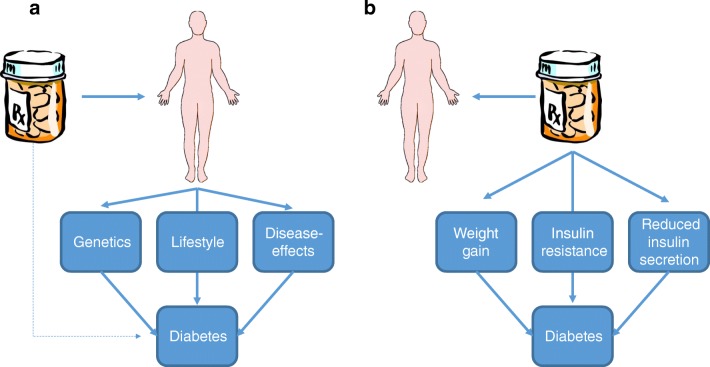
The potential mechanisms by which antipsychotics are associated with an increased risk of diabetes. **a** Antipsychotics are used in individuals with severe mental illness who are increased risk of diabetes because of genetic, lifestyle and disease effects. In this scenario, the relationship between the antipsychotics and diabetes is not causal. **b** Antipsychotics increase the risk of diabetes by increasing body weight, inducing insulin resistance and impairing insulin secretion in a causal manner. The development of diabetes in any one individual is likely to be a combination of both possibilities

As genetics, environmental and disease-specific effects may all contribute to the increased risk of diabetes in people with psychosis, it is difficult to assess the degree, if any, to which an antipsychotic has contributed to hyperglycaemia [9]. Metabolic abnormalities are observed in people prior to any treatment as shown by a recent systematic review of 16 case-control studies including 731 people with first-episode psychosis [10•]. Fasting and post-glucose challenge plasma glucose values were increased in association with elevated insulin concentration and measures of insulin resistance. As many of those developing diabetes have other risk factors, the link between antipsychotic and diabetes could be one of association rather than causation (Fig. 1).

Much of the early evidence relied on pharmaco-epidemiological studies. Although these studied large numbers of people taking antipsychotics and had the power to detect a small risk of diabetes, they are prone to a number of biases and potential confounders [9]. These include poor data quality, screening bias (people taking a particular antipsychotic are more likely to be tested for diabetes), failure to adjust for other potentially more important risk factors for diabetes and prescriber bias including channelling bias (particular antipsychotics are more likely to be used in people with a higher risk of diabetes). The quality of metabolic side effect reporting in randomised clinical trials has improved over the last decade but caution is still needed because most trials are underpowered to examine changes in glucose and are often of too short a duration given the long natural history of diabetes.

## Clinical Evidence

Publications over the last 5 years largely confirm what we previously knew rather than adding substantially new insights; nevertheless, the studies have extended the reach by assessing longer duration of antipsychotic use and new populations and have added information on newer antipsychotics.

The rates of diabetes are higher among people taking either first-generation or second-generation antipsychotics than the general population. There is a rapid rise in diabetes after treatment initiation suggesting that the antipsychotics may be implicated. A large meta-analysis of 438,245 people with severe mental illness found that the prevalence of type 2 diabetes in people prior to antipsychotic treatment was 2.9% and increased to an overall prevalence of 11.3% among those receiving treatment [11]. The prevalence of diabetes was higher in women and those with multiple episode of psychosis.

Studies, including randomised controlled trials, comparing different antipsychotics indicate that the risk of developing diabetes differs between antipsychotics. There is a consistently higher reported risk of diabetes in people taking olanzapine or clozapine with the lowest risks associated with aripiprazole. In the study cited in the previous paragraph, all antipsychotics, except aripiprazole and amisulpride, were associated with a higher prevalence of diabetes [11]. The differences between second-generation antipsychotics were also highlighted in a systematic review of 15 population-based studies [12]. An increased risk of diabetes was found in one of the two aripiprazole studies, three of four clozapine studies, seven of nine olanzapine studies, five of nine risperidone studies, three of six quetiapine studies and both ziprasidone studies.

Studies in people who are naïve to antipsychotic treatment are particularly relevant because these individuals have no residual effect of previous exposure to antipsychotics. A study using data from the Danish Central Psychiatric Research Registry followed 7139 people with first-episode psychosis for 6.6 years, of whom 307 individuals developed diabetes [13]. Initial treatment with olanzapine or mid-potency first-generation antipsychotics (e.g. zuclopenthixol, prochlorperazine and perphenazine) was associated with a shorter time to the onset of the diabetes. However, although the hazard ratio for olanzapine was 1.41, this was smaller than the hazard ratio for the use of antihypertensive (HR 1.87) or lipid-lowering medication (HR 4.67), implying that other more traditional risk factors for diabetes may be more important than antipsychotic treatment. The report also found that the development of diabetes was associated with current use of olanzapine and clozapine while aripiprazole was associated with a lower risk.

The lower risk of diabetes with aripiprazole was observed in two other real world comparisons of aripiprazole with other second-generation antipsychotics [14]. The first also utilised Danish registry data and compared the rates of diabetes in 345,937 who had purchased antipsychotics with nearly 1.5 million people not taking antipsychotic therapy [15]. Over a 10-year follow-up, over 50,000 people developed diabetes. The incidence of diabetes was increased among people taking either first- or second-generation antipsychotics by 53% and 32% respectively. The risk of diabetes increased with greater exposure to antipsychotics; for those cashing more than 40 prescriptions, the rate ratio increased to 2.04 and 1.81 for first- or second-generation antipsychotics respectively. Diabetes risk also increased with the number of antipsychotics prescribed. Incident diabetes varied between different second-generation antipsychotic drugs, with the highest risk observed with ziprasidone and sertindole. There was no increase in incident diabetes following treatment with amisulpride, quetiapine and aripiprazole.

The second study involved 55,287 people taking antipsychotics who were enrolled with Kaiser Permanente Health Plan, HealthCore Integrated Research Network or PharMetrics [16]. During the 3.25-year follow-up, 357 people developed newly treated diabetes. Compared with first-generation antipsychotics, people using olanzapine had a 71% increased risk of diabetes, while no increased risk was seen for aripiprazole, quetiapine, risperidone or ziprasidone. The hazard ratio was greatest for clozapine (HR 2.58) but this was not statistically significant because the numbers taking clozapine were so small.

Although randomised controlled trials provide the best evidence of a drug effect, most antipsychotics trials are under-powered to demonstrate a difference in incident diabetes; however, as blood glucose reporting has improved, it is now possible to examine whether differences in glucose occur between antipsychotics.

In 2010, Rummel-Kluge et al. published a systematic review of 48 studies comparing the head-to-head metabolic side effects of second-generation antipsychotics [17]. The use of olanzapine led to a greater increase in glucose than amisulpride (mean difference 0.4 mmol/L), aripiprazole (0.2 mmol/L), quetiapine (0.5 mmol/L), risperidone (0.3 mmol/L) and ziprasidone (0.5 mmol/L). Clozapine produced a similar increase in glucose to olanzapine but no other differences between drugs were reported. Although these differences are small, most studies lasted less than 6 months and many were shorter than 10 weeks and the changes with longer duration of treatment are unknown. The study with the longest treatment duration studied five antipsychotics over 78 weeks; the exposure-adjusted increase in baseline glucose varied from 0.2 mmol/L for ziprasidone to 0.8 mmol/L for olanzapine with intermediate changes for quetiapine (0.4 mmol/L), risperidone (0.4 mmol/L) and perphenazine (0.3 mmol/L).

More recently, Zhang et al. have published a network meta-analysis of 47 studies with 114 comparisons [18••]. Only olanzapine was associated with higher glucose levels compared with a placebo (0.2 mmol/L). Furthermore, olanzapine was associated with a significantly greater change in glucose than ziprasidone (0.3 mmol/L), lurasidone (0.3 mmol/L) or risperidone (0.2 mmol/L). Ziprasidone, lurasidone and aripiprazole were associated with the least effect. All studies lasted less than 1 year but when the studies were assessed according to duration, differences between antipsychotics were only found in studies lasting longer than 12 weeks.

Higher antipsychotic doses are associated with a higher risk of diabetes. In a study of nearly 50,000 people prescribed antipsychotics for more than 45 days and followed for 2.5 years, there was a dose-dependent increase in the risk of diabetes in those taking olanzapine, with the rate being 42% higher in the top third of doses compared with the lowest tertile [19]. Quetiapine and risperidone users in the top dose tertile had an increased diabetes risk but there was no increased risk with lower doses. There was no increased risk in individuals taking aripiprazole or ziprasidone.

While the evidence is clear that antipsychotic usage is associated with an increased risk of diabetes and there is a hierarchy of risk between antipsychotics, there is less consistency about the magnitude of this risk. The reported risk ratios are highly variable ranging up to a 33-fold increase but often with wide confidence intervals; however, typically the relative risks are less than two [12]. The incidence rates of diabetes are generally low, usually less than 1%, meaning that the absolute risk of any one individual is small [13, 15].

While the risk of developing diabetes-related microvascular complications is higher in people taking antipsychotic medication, it is unknown whether this differs between antipsychotics [20]. As well as the adverse effect on glucose and body weight, antipsychotics also worsen the lipid profile and so it may be expected that the incidence of macrovascular disease would differ between antipsychotics. Epidemiological studies, however, have not demonstrated a higher risk of cardiovascular disease and mortality in people taking second-generation antipsychotics [21], and, in particular, clozapine or olanzapine [22].

### Adolescents and Young Adults

The relative risks for diabetes appear to be higher in adolescents and young adults. A meta-analysis of 13 studies involving 185,105 people found that the risk of type 2 diabetes was increased by 5.72-fold but the incidence rate was low (3 per 1000 patient-years) [23•]. The risk increased in boys, with longer treatment duration, and with the use of olanzapine.

### Gestational Diabetes

There is an emerging literature on the effect of antipsychotics on gestational diabetes. A systematic review of 10 studies found that the prevalence of gestational diabetes varied widely from 2.6 to 22% in women taking antipsychotics compared with 0.9 to 10.7% in women taking no medication; however, this difference was statistically significant in comparative studies [24]. Another study reporting data from over 1.5 million women, of whom over 10,000 were taking antipsychotics during the first half of pregnancy, found that there was a 61% and 28% increased risk of gestational diabetes in women taking olanzapine or quetiapine respectively [25••]. No increased risk was seen in women taking aripiprazole, ziprasidone and risperidone or in women who discontinued antipsychotics.

## Mechanisms

The type of diabetes that occurs in the vast majority of people taking antipsychotics is type 2 diabetes, and there is no evidence that antipsychotics alter islet autoimmunity or induce type 1 diabetes [26]. Initially it was believed that antipsychotics increased the risk of diabetes by promoting weight gain but there is also evidence that antipsychotics directly decrease insulin sensitivity and insulin secretory capacity (Fig. 1).

### Insulin Resistance

#### Weight Gain

Nutrient excess is a key driver of insulin resistance. In the adipocyte, uncontrolled lipolysis increases circulating free fatty acids, which stimulate gluconeogenesis and induce hepatic and muscle insulin resistance through the accumulation of specific lipid intermediates, such as diacylglycerol. The risk of type 2 diabetes increases markedly with increasing weight and body mass index with obesity accounting for 80–85% of the overall risk of type 2 diabetes. In Western Europe and North America, the vast majority of adults with type 2 diabetes are overweight or obese.

Obesity rates are increased 2–3-fold among people with severe mental illness [27]. This occurs early in the natural history of schizophrenia with a significant proportion of people with first-episode psychosis being overweight prior to antipsychotic initiation. Substantial and rapid weight gain often occurs within 6–8 weeks after treatment initiation [28] and continues in the longer term albeit at a slower rate [29].

Unhealthy food choices, physical inactivity and social deprivation as well as disease-specific mechanisms all contribute to obesity in people with severe mental illness but the use of antipsychotics is the most important factor related to weight gain. These drugs are highly obesogenic drugs and more than 7% weight gain occurs in 15–72% of people taking second-generation antipsychotics [30].

Antipsychotics induce weight gain largely through increased appetite and food intake [27]. The regulation of body weight is highly complex and results from the co-ordination of a wide variety of hormonal and nervous signals from the gastrointestinal tract, pancreas, adipose tissue and central nervous system to the hypothalamus, which in turn regulates feeding behaviour and metabolism. Many neuropeptides are involved in this process but include several that are affected by antipsychotics (Table 1) [31•]. Serotonin acting through the 5-hydroxtryptamine_2C_ (5-HT_2C_) receptor stimulates anorexigenic proopiomelanocortin (POMC) neurons and decreases appetite [32]. Histamine acting through H_1_ receptors reduces the hypothalamic production of AMP-activated protein kinase (AMPK) [33]; this enzyme acts a cellular ‘fuel gauge’ and when activated stimulates appetite. Dopamine is involved in reward pathways and hedonistic control of appetite [34]. Antipsychotics appear to increase appetite by inhibiting these receptors. Downstream effects include decreased POMC neuron activation and increased AMPK activity.

Over the last decade, the gut microbiome has been identified as an important factor in the pathogenesis of obesity and other metabolic diseases. The chronic administration of olanzapine leads to an alteration of the microbiome in rats while the co-administration of antibiotics can attenuate olanzapine-induced weight gain [35].

Antipsychotics may further worsen weight gain by reducing energy expenditure. Many antipsychotics are sedatives and reduce voluntary movement. Whether antipsychotics alter resting energy expenditure through altered thermogenesis is uncertain with conflicting results in the literature [32, 36].

#### Direct Effect on Insulin Resistance

The mechanisms underlying the development of insulin resistance are not fully understood and may occur at many levels of insulin signalling. However, the primary defect appears to be a markedly impaired ability to phosphorylate the post-receptor insulin receptor substrate, IRS-1, which in turn leads to downregulation of a number of intracellular downstream pathways including the phosphoinositide 3-kinase (PI3K)/protein kinase B or Akt pathway.

In vivo human studies have demonstrated a worsening of insulin resistance with olanzapine and aripiprazole independent of any change in weight or body mass, suggesting that antipsychotics have a direct effect on insulin sensitivity [31•]. Rodent experiments complement these findings and show that olanzapine, clozapine, risperidone and haloperidol cause dose- and time-dependent changes in insulin sensitivity. Cell culture experiments have shown that olanzapine, but not amisulpride, inhibits the Akt pathway in myotubes leading to a decrease glycogen content [37]. Olanzapine also attenuates insulin-induced phosphorylation of insulin-like growth factor receptor and IRS1 in fibroblasts through a mechanism involving membrane-associated mammalian neuraminidase-3 (Neu3) and Neu 1 sialidase [38]. Similarly, clozapine reduces insulin-stimulated glucose uptake in PC12 and L6 cells, by inhibiting IRS-1 phosphorylation and the Akt pathway [39]. Inhibition of the Akt pathway by antipsychotics has also been observed in T cells and glioblastoma cells [40, 41].

### Effects on Pancreatic Islet β Cells

In type 2 diabetes, insulin secretion is reduced and abnormalities in β cell function are a major determinant of the rate of diabetes progression. People with severe mental illness are 10 times more likely to develop diabetic ketoacidosis as a first presentation of diabetes than the general population and the rates of diabetic ketoacidosis are 30-fold higher in people with existing diabetes and severe mental illness compared those with diabetes alone [42, 43].

One series identified 23 cases with a median time to diabetic ketoacidosis of 5 months after antipsychotic initiation. Although 9 individuals were subsequently confirmed to have type 1 diabetes, antipsychotics were implicated in the remainder. These included olanzapine (*n* = 9), aripiprazole (*n* = 6), risperidone (*n* = 6), clozapine (*n* = 3) and quetiapine (*n* = 1) [44]. A further literature review found 65 case reports of diabetic ketoacidosis, involving monotherapy with olanzapine (*n* = 26), clozapine (*n* = 17), risperidone (*n* = 6), aripiprazole (*n* = 6), quetiapine (*n* = 3) and polypharmacy (*n* = 7) [45]. In five cases, the same antipsychotic was re-started after recovery with subsequent hyperglycaemia in four cases. By contrast, in the majority of the remaining cases, a different antipsychotic was substituted without a recurrence of the metabolic disturbance.

As diabetic ketoacidosis only occurs in situations of severe insulin deficiency, this implies that antipsychotics reduce insulin secretion, either through a toxic effect on the pancreatic β cells or pharmacological action that disrupts normal insulin secretion.

Animal and cell culture experiments have shown the olanzapine and clozapine impair insulin secretion [1]. It is likely that these effects are mediated through several receptors as dopaminergic, serotonergic, adrenergic and muscarinic receptors all have a role in regulating insulin secretion (Table 1). Antagonism of the dopamine D_2_ receptors blunts glucose-stimulated insulin release while blockade of the 5-HT_1a_ and M_3_ muscarinic receptors decreases the responsiveness of pancreatic β cell to changes in blood glucose. Blocking α_2_ adrenergic receptors increases basal insulin secretion. In addition to the pharmacological effects, cell culture experiments have shown that antipsychotics increase apoptosis of the β cells.

## Mitigating the Effects of Antipsychotics on Diabetes

Antipsychotics are the mainstay of the treatment of severe mental illness. Initially antipsychotics are needed to manage the acute psychosis but in the longer term, these drugs are necessary to prevent relapse. Treatment is associated with a lower risk of hospitalisation, suicide and all-cause mortality [22, 46]. Nevertheless, the risk of diabetes needs to be considered and strategies developed to prevent diabetes, screen and diagnose diabetes and manage diabetes if it occurs.

### Prevention of Diabetes

#### Lifestyle

Randomised controlled trials in the general population have demonstrated the effectiveness of lifestyle intervention to prevent diabetes [47–51]. No diabetes prevention studies have been undertaken in people taking antipsychotics, but there is a growing literature on the use of lifestyle interventions to prevent weight gain or manage obesity. Short-term studies lasting less than 6 months report significant weight loss with lifestyle interventions with one meta-analysis of non-pharmacological interventions showing a mean reduction of 3.1 kg over a period of 8–24 weeks [52]. The results of longer term studies are less consistent with a more recent meta-analysis describing significant weight loss in only two of six studies with interventions lasting longer than a year [53]. Interventions that are more intensive appear to yield greater weight loss but whether these could be implemented into routine clinical practice is debatable [54–57]. Furthermore, it seems that lifestyle interventions may be less effective in people with schizophrenia compared with other severe mental illnesses [54–57]. These findings also have implications for lifestyle modification following a diagnosis of diabetes. Recently, the DiRECT trial has shown that intensive weight management can lead to diabetes remission, but whether this intervention could be delivered successfully to people with severe mental illness is unknown [58]. While lifestyle change is clearly important for people with severe mental illness, further work is needed to understand the optimal way to deliver lifestyle interventions.

#### Pharmacotherapy

Metformin and orlistat reduce incident diabetes in the general population [51, 59]. Again, the effects of these drugs on the risk of diabetes in people taking antipsychotics have not been studied but a meta-analysis of 12 studies involving 743 people treated with antipsychotics showed that metformin lead to a mean 3.3-kg reduction in body weight over 3–6 months. Insulin resistance also improved but there was no change in fasting glucose [60].

Although orlistat causes a modest weight reduction in people prescribed either clozapine or olanzapine [61], this medication is difficult to take because of the high incidence of gastro-intestinal side effects. Glucagon-like peptide-1 (GLP-1) receptor agonists promote weight loss in people with diabetes and liraglutide is also licensed as an obesity medication [62]. Three trials using this drug class have been completed in people taking antipsychotics. Although no effects on body weight were seen in one of the two trials of exenatide [62], the other showed a mean weight loss of 5.3 kg and a small reduction in HbA_1c_ after 12-week treatment [63]. Similarly, liraglutide, at a maximum dose of 1.8 mg, caused a 5.3-kg reduction in weight together with improved glucose tolerance [64]. A trial of 3 mg liraglutide in people with schizophrenia is currently ongoing (Universal Trial Number U1111-1203-0068; EudraCT: 2017-004064-35).

Several studies suggest that switching to an antipsychotic with a lower propensity for weight gain may result in weight loss or attenuation of weight gain although any decision to change antipsychotic must balance the risk of relapse of the core psychotic symptoms [65]. Another approach is adjunctive aripiprazole as three randomised controlled trials have shown that adding aripiprazole to either clozapine or olanzapine results in weight loss of just over 2 kg [66].

### Screening and Diagnosis of Diabetes in People Taking Antipsychotics

Screening for diabetes is recommended at treatment initiation or when treatment is changed [67,68••,^69^]. Further testing should be performed 3–4 months later to identify the small number of people who develop diabetes rapidly after starting antipsychotic treatment and annually thereafter. The most convenient test is HbA_1c_ but this may be falsely negative if there is a rapid onset of hyperglycaemia as sometimes happens after beginning antipsychotic treatment. Fasting or random glucose tests are acceptable alternatives. Despite clear guidance and potential benefit, many people taking antipsychotics are not screened regularly and further work is needed to embed this simple measure into routine clinical practice [70, 71]. Many community psychiatric settings do not have the facilities to obtain laboratory blood samples and so point-of-care testing of glucose, and ketones if required, may increase the uptake of screening for diabetes.

### Management of those with Diabetes

Diabetes should be generally managed in people taking antipsychotics as for the general population. Given the high prevalence of obesity, the use of agents that cause weight loss may some advantages. Although the safety of GLP-1 receptor agonists has been evaluated in three trials in people taking antipsychotics, similar trials have not been undertaken with SGLT2 inhibitors. Although a rare adverse effect, SGLT2 inhibitor treatment may precipitate euglycaemic diabetic ketoacidosis and caution is needed in people taking antipsychotics as these may also induce ketoacidosis. However, this risk should be balanced with the potential cardiovascular benefits of this class of drugs.

## Conclusion

Antipsychotics play an essential part of the management of people with severe mental illness; however, this comes at the cost of an increased risk of weight gain and, for a minority, diabetes. Antipsychotics exert their therapeutic effect, predominantly through antagonism of the dopamine D_2_ receptor; however, many also exert effects at serotonin, histamine and adrenergic receptors. These receptors are also involved with the regulation of body weight and intermediate metabolism, as well as insulin secretion, and may be responsible for the adverse effects of weight gain and diabetes. There is a hierarchy of risk with different antipsychotics that is explained in part by the different patterns of receptor binding.

Antipsychotics reduce hospitalisation and death and so in many situations, these drugs cannot be stopped but measures can be taken to mitigate against the adverse effects including lifestyle or drug interventions to reduce weight gain. If diabetes occurs, this should be diagnosed in a timely manner and managed effectively.
